# Responsibility problems for criminal justice

**DOI:** 10.3389/fpsyg.2014.00821

**Published:** 2014-07-29

**Authors:** Sofia M. I. Jeppsson

**Affiliations:** Department of Philosophy, Stockholm UniversityStockholm, Sweden

**Keywords:** moral responsibility, criminal justice, compatibilism, negative retributivism, desert, retributivist side-constraints, mitigating circumstances

It has been argued that empirical science undermines the claim that people can deserve punishment, and that the criminal justice system therefore ought to be radically reformed. Such arguments lose their force if moral responsibility and desert do not depend on what caused the action, but on the agent's choice. We solve one problem for the justification of the criminal justice system, but create another one; if moral responsibility depends on the offender's choice, finding out to what extent she was responsible might be very difficult.

Our common practice of holding each other responsible for our actions contains elements of character evaluation and pragmatism, i.e., encouraging some behaviors and discouraging others. We also have the idea that people can be morally responsible for what they do in the sense of *deserving* to be praised for exemplary actions and blamed for bad ones—and even punished, if the action was bad enough. Many philosophers and legal theorists who believe that the primary goal of the criminal justice system ought to be crime prevention rather than the dealing out of just deserts, still argue that the offenders' desert ought to serve as a restriction on what we are allowed to do in the name of crime prevention; no one must be given more punishment than she deserves (e.g., von Hirsch, 1992; Lippke, 2014). Since no system is perfect, it is inevitable that this principle will *sometimes* be violated, but we ought to strive for a system that allows us to consistently approximate this ideal. However, if no one were morally responsible for anything, *all* punishments would be undeserved, and the criminal justice system difficult to ethically justify.

Some philosophers and scientists do argue for the non-existence of moral responsibility and desert, roughly along the following lines: Whether an offender was morally responsible for what she did depends on how her action was caused. If it was caused by events beyond her control, she lacks moral responsibility for it. Therefore, she does not deserve to be punished if her crime were caused by, e.g., psychosis, someone slipping a drug into her drink, or someone making an irresistible threat toward her. However, *all* crimes are ultimately caused by events beyond the offender's control (e.g., non-conscious events in her brain, genes and environment). Therefore, no one ever deserves to be punished (Pereboom, 2001; Strawson, 2002; Greene and Cohen, 2004; Harris, 2012). If these philosophers are right, any system for dealing with criminals resembling the current one might be ethically unjustifiable.

However, this whole argument fails if we deny the initial premise that moral responsibility for an action depends on how it was caused.

Some philosophers of law and legal theorists do deny that premise; Morse (2013) and Moore (1997) argue that the law as it stands permits punishing offenders when they are capable of making choices for reasons. Furthermore, there is nothing wrong with the law on this point; the thesis that offenders can deserve punishment for what they have chosen to do can be defended by philosophical argument. Many Kantian philosophers argue that actions can be viewed from two different perspectives; a theoretical one, where we explain why someone did what she did by pointing at causes, and a practical one, where we focus on her choice and her reasons for taking one option rather than another. The claims we make from those different perspectives do not contradict each other. I might have chosen to become a philosopher for the reason that I found philosophy interesting. If a scientist were to discover the neurological causation of interest, it would still be true that I chose a philosophy career for the reason I did. Since morality is concerned with making the right choices for the right reasons, moral judgments ought to be made from a practical perspective. Whether someone was morally responsible for an action and deserves to be praised, blamed, or punished depends on the choice she made, not the underlying causes (Korsgaard, 1996; Bok, 1998; Dworkin, 2011, pp. 224 and 462; Jeppsson, 2012). I call this thesis “Practical Perspective Compatibilism,” or PPC.

According to PPC, many offenders are morally responsible for what they did, and would thus deserve to be punished, since many offenders chose to commit a crime. PPC can also explain why some psychotic, drugged or seriously threatened offenders ought to be excused: in these states, they might very well be bereft of choice. Alternatively, in the case of a serious threat, the offender might have consciously chosen to do the least bad thing in a terrible situation; even if she were morally responsible for this choice we might judge that she did nothing *wrong* if she, e.g., stole an object because someone threatened to kill her children otherwise, and therefore she ought to go unpunished. It is evident that these excuses do not generalize to all offenders. It is still the case that many offenders choose to commit crimes, and no advancements made in neurobiology or other empirical sciences will undermine this claim. Their choices may have had causes, but they were still choices. PPC thus solves one problem for the ethical justification of criminal justice, but it creates another one; if moral responsibility depends on the offender's choice, finding out to what *extent* she was responsible might be very difficult.

Drawing the line between agents who deserve some kind of punishment for committing a crime and those who ought to be completely excused might not be too difficult in most cases (Moore, 1997, p. 112; Kenny, 2010, pp. 392–401). But if moral responsibility and desert depend on the offender choosing actions, cases where the offender deserves less punishment due to having diminished responsibility for her crime will be difficult to judge^1^. If moral responsibility depends on the offender's choice, mitigating circumstances mitigate only insofar as they affect said choice. When the offender made less of a choice, she was less responsible (Jeppsson, 2012, pp. 58–67; Coates and Swenson, 2013). This claim is intuitively plausible. When choosing what to do, we try to find an option that we have most or at least sufficient reason to pursue, according to our own views about reasons (Jeppsson, 2012, pp. 59–60; see also Wolf, 1990, p. 31; implicit in Kapitan, 1986; Pereboom, 2008). (This assumption is not supposed to be controversial, since “our own views about reasons” may encompass a wide range of views.) We often consider only a few options, or immediately choose what to do without considering alternative actions at all, because it is immediately obvious to us that this option is at least good enough. But occasionally agents fail to consider options that were actually superior, according to the agents' own views about reasons, to the option they picked, merely because these other options somehow did not strike them as real alternatives. They fail to fully choose what to do. If moral responsibility depends on choice, someone who did not fully choose is plausibly less than fully responsible.

The PPC theory of diminished responsibility thus has the resources to explain, not only why some psychotic, drugged or seriously threatened offenders ought to be completely excused, but also the fairly common judgment that a harsh environment can constitute mitigating circumstances (e.g., Hudson, 1995, 1999). We might think that a young criminal from a run-down, high-crime neighborhood is less responsible for her crimes, and therefore less deserving of punishment, than a young criminal who had everything going for her and yet chose to commit crimes. The criminal from the bad neighborhood might have been *expected* to turn to crime; she internalized these expectations, and failed to really see honesty as an alternative, even though an honest life might have seemed preferable to her had she really thought about it. She did not fully *choose* to become a criminal (whereas her more well-to-do counterpart made an active decision to engage in crime), and therefore her responsibility is diminished. These explanations of why a harsh environment is mitigating are intuitively more plausible than anything a causality-based theory of moral responsibility can provide, since it does not generally seem to be the case that causal influences behind one's choice renders one less responsible (I am presumably fully responsible for becoming a philosopher, despite the fact that this decision was undoubtedly influenced by a number of external factors).

However, we know that similar circumstances do not affect everyone equally. It is possible that a young criminal from a run-down and high-crime neighborhood did think things through and made an informed decision to become a criminal rather than engage in honest work. It is thus possible that out of two young criminals with a similar background, committing their crimes in similar circumstances, one is fully morally responsible for what she did and therefore deserves a harsh punishment, whereas the other one has diminished moral responsibility and deserves leniency. The same thing can be said about any circumstance that is normally considered mitigating; whether it diminishes the responsibility of this particular offender or not, depends on how it affected her choice. It seems difficult, to say the least, to ascertain how much punishment offenders deserve in particular cases, if moral responsibility and desert depend on their choices.

We might try to ensure that we do not give some offenders more punishment than they deserve by adopting a generally lenient approach when sentencing (Duus-Otterström, 2013). Possibly, in order to be on the safe side, we would have to be very lenient, to an extent that seriously conflicts with the goal of crime prevention. However, there is some empirical support for the thesis that if people are led to believe that they were not really responsible for what they did, this belief makes them follow temptation rather than making active choices (Vohs and Schooler, 2008), i.e., people's belief that they lack moral responsibility might actually erode their moral responsibility. Even if we were willing to accept that there are offenders who are chronically bad at making choices and keep performing actions that they do not really believe that they have reason to do, and who therefore never come to deserve more than fairly mild punishment despite repeated crimes, a system that actually *pushed* people in that direction would certainly be a failed one.

Thus, PPC ensures that offenders can be morally responsible for their crimes and therefore deserve punishment, regardless of what neurobiology and other empirical sciences might find. But PPC also implies that finding out to what *extent* someone was responsible for what she did might be very difficult, perhaps even impossible.

## Conclusion

If moral responsibility depends on the agent's choice rather than on her action being caused in the right way, we need not worry that findings in neurobiology or other empirical sciences will undermine the claim that people can be morally responsible for what they do. This might seem like good news for the criminal justice system, insofar as it depends on the assumption that offenders can deserve to be punished for its ethical justification. However, if an offender's level of moral responsibility ultimately depends on how she chose to do what she did, finding out to what *extent* she was morally responsible for her crime, and thus how much punishment she deserves, might be difficult. If we ought not to punish anyone harder than she deserves, this is a problem that must be addressed.

### Conflict of interest statement

The author declares that the research was conducted in the absence of any commercial or financial relationships that could be construed as a potential conflict of interest.

## Footnotes

^1^Even if we set aside the acknowledged problem of matching up appropriate punishments with crimes (Duff, 1986, p. 280; von Hirsch, 1992).
